# A Texas Home for Epileptics

**Published:** 1895-11

**Authors:** 


A TEXAS HOME FOR EPILEPTICS: A GOOD SUGGESTION — An eminent physician of Southern Texas, a man of large experience in the practice of medicine, writes to the JOURNAL to inquire how many epileptics there are in Texas, and says:

"Something should be done for this unfortunate class, the most miserable on earth. By the time they reach middle age they have, as a rule, spent their all in vainly trying to be cured. After that, the poor house, the jail or the insane asylum is their lot. There is no occupation,—no position in life, however humble, open to them; nobody wants them. The great State of

Texas must do something for them! The State of New York recognized the necessity of caring for this unfortunate class, and by legislative enactment purchased a large tract of land and built cottage homes for the epileptics. I am not informed as to the results, and would be glad to get hold of a report on the subject. Why can not Texas do likewise? Let her purchase, or set aside from the vast public domain, a suitable tract of land, establish farms, workshops, etc., and place in charge,—not necessarily a doctor,—but some man properly qualified for the duties that would devolve upon him, and with a salary that would justify him in giving his entire time and attention to it; let the appointment be for lifetime, and removal except for cause, impossible. There are many cases of epilepsy amenable to cure, many can be relieved, and perhaps all, bettered, mentally, morally and physically, by proper medical treatment, hygiene and environment, and the misery of their sad lot greatly ameliorated. In a few years such an institution could be made self-sustaining, if not a source of revenue to the State.

Now, my dear 'Red-Back' JOURNAL, you may depend upon it, Texas *shall do* this! May be, not in your life time nor mine, but it will, must, *shall* come. Can not the enterprising and indomitable exponent of medical thought and progress in Texas, the popular 'Red-Back' red hot TEXAS MEDICAL JOURNAL start the ball in motion? In my humble opinion, the State Medical Association should take up the subject, and memorialize the legislature to the end above indicated.'' H. A. T.

The above is a sensible letter, and the suggestion is good and timely. The JOURNAL will do whatever may lie in its power to further the scheme.

The first thing to be done in the above connection, in the JOURNAL'S opinion, is to secure the passage of a law regulating marriage, and prohibiting matrimony between persons either of whom is hereditarily tainted with epilepsy, consumption, syphilis, cancer or insanity; between paupers also; and the privilege should be denied to all who have ever been convicted of felony. Before any attempt is made to care for those now on hand, steps should be taken to arrest the propagation of epileptics; in fact, all defectives who in time become wards of the State and a burden to tax payers.

The course pursued by all civilized people in caring for the insane, the blind, the deaf and dumb, is calculated to foster and develop these respective classes and insure a perpetuity, with a steady increase in numbers. Such course, tenderly caring for them in palatial buildings, surrounded by every comfort, is perhaps in accordance with the dictates of humanity; but in the interest of society, it is all wrong; it defeats the law of elimination, and tends to make the unfit survive.

If the State have the right to regulate matrimony in any degree (and we know that there are certain conditions affixed to the privilege, e. g., persons shall not marry within a certain degree of kinship—consanguineous relationship), it certainly should have the power to go further, and in the interest of the State and of society, and for the protection of tax payers, should forbid the marriage of persons who are subject by inheritance of predisposition either to disease or crime; and while making provision for the defectives which our fathers bequeathed to us, through, some think, mistaken notions of benevolence, we should *cut off the supply*, and prevent the perpetuity, which is sure to follow, so long as anybody can marry. It is not uncommon that a lunatic is discharged ''cured'' or ''improved,'' and there is nothing to prevent his contracting relations that will almost surely secure to the next generation a full crop of his kind,--children born with a predisposition to insanity; and we have known paupers, on the poor farm, to be united in the ''holy bonds of matrimony,'' with the sanction of the law and the blessing of the church, all unmindful of, or with a reckless indifference to the fact, that in permitting it the State is increasing the burden upon tax payers, and insuring a succession of paupers to the next generation.

When a man has demonstrated his inability to feed and cloth himself, and becomes a burden on the State, unless he be stricken by age, or is subject for one of the other charities, it would be the part of wisdom and good policy to *castrate* him; certainly to deny to him the privilege of a progeny. The same remark applies to those who by their evil deeds have forfeited all other rights as citizens—the convicted felon. We were asked recently, ''what should be done with the defectives of all classes now a charge to the State?'' Our interlocutor evidently regarding us as a very uncharitable or unchristian man because we said these unfortunates should not be allowed to procreate; we replied, ''The fittest survive, if nature can have her way. We do all we can to defeat nature. Care for all now on hand, and in the course of time they will die out; see to it that they do not bequeath to the next generation a progeny of like kind, only increased in numbers.''

Therefore, we repeat, the first duty of the State is to *cut off the supply* of insane, deaf and dumb, blind (congenital), paupers and criminals, some by prohibiting marriage, some by asexualization.

''But,'' said our interviewer, ''as you forbid marriage you increase prostitution.''

Perhaps so; but is it not the least of two evils, seeing, that, as a rule, prostitutes do not breed; nature asserts herself here, and, as in the case of hybrids, puts a stop to fecundation.

With the proviso that we are not to have a perpetuity of epileptics, insane, deaf and dumb, paupers and criminals, we say yes, build insane asylums and jails for the present generation, and farms and shops for the epileptics. Care for them according to the dictates of humanity; but—stop the breed!

